# Influence of Coactors on Saccadic and Manual Responses

**DOI:** 10.1177/2041669517692814

**Published:** 2017-02-01

**Authors:** Manuel Oliva, Diederick C. Niehorster, Halszka Jarodzka, Kenneth Holmqvist

**Affiliations:** Department of Cognitive Science, Lund University, Sweden; Humanities Laboratory & Department of Psychology, Lund University, Sweden & Institute for Psychology, University of Muenster, Germany; Welten Institute, Open University of the Netherlands, The Netherlands; Humanities Laboratory, Lund University, Sweden & UPSET, NWU Vaal, South Africa

**Keywords:** Attention, cognition, divided attention/resource competition, endogenous/exogenous, eye movements, social cognition

## Abstract

Two experiments were conducted to investigate the effects of coaction on saccadic and manual responses. Participants performed the experiments either in a solitary condition or in a group of coactors who performed the same tasks at the same time. In Experiment 1, participants completed a pro- and antisaccade task where they were required to make saccades towards (prosaccades) or away (antisaccades) from a peripheral visual stimulus. In Experiment 2, participants performed a visual discrimination task that required both making a saccade towards a peripheral stimulus and making a manual response in reaction to the stimulus’s orientation. The results showed that performance of stimulus-driven responses was independent of the social context, while volitionally controlled responses were delayed by the presence of coactors. These findings are in line with studies assessing the effect of attentional load on saccadic control during dual-task paradigms. In particular, antisaccades – but not prosaccades – were influenced by the type of social context. Additionally, the number of coactors present in the group had a moderating effect on both saccadic and manual responses. The results support an attentional view of social influences.

## Introduction

Imagine writing an exam in a large hall where row after row of other students take the same test. You hardly look at each other, let alone talk, but you are likely to think about their progress compared to your own, and you experience how their presence affects your thoughts and actions. One can think of many situations where our behaviour is influenced by the type of social context we are in. Indeed, research has shown that even the mere presence of another person can affect our attention and actions (Richardson et al., 2012; Zajonc, 1965). A meta-analysis of 241 studies found that the presence of others improves the speed at which simple tasks are completed but decreases the speed for complex tasks (Bond & Titus, 1983). Although these social effects have been thoroughly studied, their causes are still debated (Guerin, 2010). Explanations of how social presence affects task performance often refer to attentional and cognitive processes, so in this study we set out to test two major social theories with eye movement paradigms that specifically reveal attentional and cognitive processes.

The simple presence of other individuals can lead to either an increase or decrease in task performance of participants compared to when they complete the same tasks but in solitary conditions. This is referred to as the social facilitation–inhibition effect. Two major theories have been proposed to explain its causes. First, Zajonc’s (1965) Activation Theory postulates that the presence of other people increases the individual’s arousal and drive, and that this, in turn, shifts the likelihood towards displaying automatic, well-learned responses rather than cognitively controlled responses. In consequence, social presence improves performance on simple tasks requiring automatic responses but reduces performance on tasks that require cognitively mediated responses.

Secondly, Baron (1986) and Sanders, Baron, and Moore (1978) provide an alternative theory for social facilitation and inhibition, named the distraction-conflict theory. This theory is based on the view that attention is a limited resource (Broadbent, 1971; Cohen & Spacapan, 1978; Kahneman, 1973) and holds that the presence of other people competes with the task at hand for attentional resources. If individuals additionally attend to the social environment instead of exclusively attending to the task, attentional resources will be redirected away from the task. This competition for attentional resources has been shown to lead to a focusing of attention on information central to the task (Cohen & Spacapan, 1978; Easterbrook, 1959; Geen, 1976). On the one hand, such a narrowing of attention can improve performance by increasing the focus on task-relevant information and limiting the interference from distractors. For example, social presence reduced interference in a Stroop task (Huguet, Galvaing, Monteil, & Dumas, 1999) and an illusory conjunction task (Muller, Atzeni, & Butera, 2004). On the other hand, the distraction-conflict theory predicts that attentional focusing will impair performance on tasks that require the use of a larger range of information (Baron, 1986). In this study, we test these predictions specifically for visual attention and the extent of the functional visual field.

### Previous Studies of Eye Movements Under Social Presence

In recent years, eye movement research has increasingly been conducted in social settings such as supermarkets (Gidlöf, Wallin, Dewhurst, & Holmqvist, 2013), cars (Recarte & Nunes, 2003) and psychiatric clinics (Hutton & Ettinger, 2006; Munoz, Armstrong, Hampton, & Moore, 2003), where other individuals might be present besides the participant or patient. As such, researchers have recently begun to investigate how social presence influences eye movements. For instance, in situations where there is potential for social interaction, attention to social stimuli changes compared to solitary experimental conditions (e.g., Foulsham, Walker, & Kingstone, 2011). The possibility of interaction also changes the extent to which participants follow each other’s gaze to stimuli in the environment (Gallup, Chong, Kacelnik, Krebs, & Couzin, 2014). More importantly, a recent study (Richardson et al., 2012) found that even the mere presence of another person can influence attention allocation. Richardson et al. (2012) seated pairs of participants facing in opposite directions and showed them sets of pictures with positive and negative emotional valence. Although the individuals did not interact with each other, the belief that they were looking at the same stimuli at the same time shifted participants’ gazes towards the more negatively valenced pictures, compared to when they believed that their partner was engaged in a different task.

Previous research on eye movements and social presence has focused on social attention and the perceptual function of exchanging and sharing information with others. In contrast, our aim is to explore whether the presence of others affects the attentional state of individuals in a way that can be traced through eye movement paradigms.

### Saccades and Attention

The presence of others has been shown to influence individuals’ attention and action (Baron, 1986; Zajonc, 1965). Therefore, a possible avenue for the study of social presence might be provided by eye movements, which are intimately related to attentional processes (Hoffman & Subramaniam, 1995; Walker, Deubel, Schneider, & Findlay, 1997). In particular, saccades are rapid eye movements that bring the fovea onto a target of interest. This process can be driven by stimuli in the environment, in which case the saccade responds to an automatic, bottom-up saccadic program, or the saccade can be regulated in a topdown fashion, such that volitional control is used to attend to targets that are in line with task-relevant goals of the observer (Buschman & Miller, 2007; Theeuwes, 2010; Trappenberg, Dorris, Munoz, & Klein, 2001). The balance between controlled and automatic behaviour is regulated by cortical areas that have the ability to inhibit automatic responses in favour of controlled responses. Neurophysiological experiments have shown that the superior colliculus mediates bottom-up, reflexive saccadic eye movements (Braun, Weber, Mergner, & Schulte-Mönting, 1992; Schiller, Sandell, & Maunsell, 1987), whereas cortical areas such as the frontal eye fields and the dorsolateral prefrontal cortex are involved in higher levels of oculomotor control (see Munoz & Everling, 2004, and Hutton & Ettinger, 2006, for reviews), including the inhibition of the superior colliculus and its automatic saccades (e.g., see review by Johnston & Everling, 2008) in favour of taskrelevant volitional saccades.

The *antisaccade* task (Hallett, 1978) was designed to investigate the mechanisms responsible for generating automatic versus controlled eye movements. In this task, the participant either makes a prosaccade towards a sudden-onset target, or an antisaccade away from it. While prosaccades are automatic eye movements (Roberts, Hager, & Heron, 1994), antisaccades require cognitive mediation, first to inhibit the automatic prosaccade, and second to plan and generate a saccade in the direction opposite to the target (Everling & Fischer, 1998; Olk & Kingstone, 2003). Patients with frontal lobe lesions or prefrontal dysfunction usually show poor antisaccade performance, with prolonged response latencies and increased direction errors, because they lack the ability to suppress the automatic saccade towards the target (Guitton, Buchtel, & Douglas, 1985; Hutton & Ettinger, 2006; Jantz, Watanabe, Everling, & Munoz, 2013; Johnston & Everling, 2008; Munoz et al., 2003). However, poor executive control in the antisaccade task is not exclusively observed in patients. Dual-task manipulations that increase attentional load also impair the performance of antisaccades, but not prosaccades, in healthy participants to levels similar to those of prefrontal patients (Baddeley, 1992; Roberts et al., 1994; Stuyven, Van der Goten, Vandierendonck, Claeys, & Crevits, 2000). Such studies suggest that increased attentional load interferes with the normal executive control that cortical areas deploy over the superior colliculus. This leads us to think that in a social context the competition for attentional resources may elicit results similar to those previously found for dual-task manipulations on saccadic control.

Programming saccades requires the processing of visual information to reach a decision as to where to move the eye (Hanes & Schall, 1996; Paré & Hanes, 2003). Neurophysiological studies, indeed, demonstrate that neural activity in the frontal eye fields and superior colliculus accumulates linearly after target appearance, and it is only when the activity reaches a critical level that a saccade is launched (Gold & Shadlen, 2000; Hanes & Schall, 1996; Paré & Hanes, 2003). Therefore, current models for saccade generation suggest that the distributions of saccade reaction times can be studied as the result of such neural decision mechanisms (Noorani, 2014; Schall & Bichot, 1998; Schall & Hanes, 1998). The LATER model (‘Linear Approach to Threshold with Ergodic Rate’; see Carpenter, 1981; Carpenter & Williams, 1995; Reddi & Carpenter, 2000) characterizes saccade latencies in terms of the rate at which information is accumulated and approaches a threshold for deciding to launch a saccade. Little is known about how social presence influences saccade programming. Here we analyse whether the change in speed for launching a saccade between social conditions is due to a shift in the decision threshold or due to a change in the rate of information accumulation.

### The Present Study

In this study, we test the effect of the presence of coactors on automatic and controlled behaviour and discuss our findings in the light of social facilitation–inhibition theories. We use paradigms that target attention and oculomotor control as seen in eye movements. For this purpose, we compared the performance of participants in two conditions, one in which the participant conducted the tasks in the presence of others who did the same task at the same time (group condition), and another condition in which participants conducted the task alone (solitary condition).

In the first experiment, participants performed an antisaccade task and we measured how pro- and antisaccades were affected by the presence of coactors. Based on the distraction-conflict theory and studies that showed the effects of attentional load on saccade generation (Stuyven et al., 2000), we expected the following: if the presence of others competes for attentional resources, antisaccade latencies should increase in the group condition whereas the rate of direction errors will not be affected by social presence. Prosaccade performance, on the other hand, will not be affected in the group condition compared to the solitary condition, as these are automated responses.

In contrast, activation theory has different predictions: because the presence of others increases the individual’s arousal, the activation theory predicts a shift towards automatic behaviour in the group condition. Therefore, in the group condition, the automatic prosaccade response is expected to become easier (latencies decrease), while the controlled antisaccade response becomes more effortful (latencies increase). For the same reason, we expect increased antisaccade direction error in the group condition, due to an increased difficulty in suppressing the automatic prosaccades.

In the second experiment, we extend our findings using a different task. Specifically, participants completed a choice reaction time task in which they had to make a saccade to a peripheral target and give a manual response to indicate the target orientation. This task involves an automatic saccade to the target, similar to the prosaccades in Experiment 1, but it additionally features a manual response, which constitutes a controlled response. Again, activation theory predicts facilitation in automatic saccades (lower latencies), but impairment in the manual responses.

Additionally, we looked at the moderating role of group size on coaction effects. We expect that bigger group sizes would increase participants’ awareness of the presence of others, which would enlarge social influence effects (see, e.g., Latane & Zipf, 1981).

## Experiment 1

In order to assess whether the presence of coactors affects the voluntary control of eye movements, we employed the antisaccade task described above. We compared performance in a solitary condition with that in a group condition. In the group condition, participants performed independently of each other but in a group setting, and all were aware that they conducted the same task at the same time.

### Methods

#### Participants

Fifty-two participants (Mean age = 26.2, standard deviation (SD) = 5.4; 18 women) were recruited via the Internet, gave informed consent and received a movie ticket for participation. All had normal or corrected-to-normal vision. Group sizes varied from two to eight participants per recording session and averaged 4.0 participants (SD = 1.8). Participants who came to the group conditions were not told during recruitment that the experiment was conducted in groups to avoid selection effects. Four participants with an antisaccade error rate higher than 50% were excluded from the analysis; eight other participants were excluded due to excessive data loss (see ‘Data analysis’). In total, 20 participants were included in each condition.

#### Design

The experiment used a 2 × 2 mixed design in which social condition (group or solitary) was varied between subjects (20 participants for each condition) and type of saccadic reaction (pro- or antisaccade) was varied within subjects.

#### Stimuli and material

Following the recommendations by Antoniades et al. (2013), the antisaccade task consisted of 120 antisaccade trials presented in three blocks of 40 trials with optional breaks in between, and two blocks of 40 prosaccades each at the beginning and end of the task. Participants had to look at a central cross presented for a random interval between 833 ms and 2333 ms. The stimulus was presented randomly either to the left or right of the central fixation point, 9.9 cm away from the centre, which corresponds to 9.4° at a distance of 65 cm. The target was a white dot with a diameter of 0.53° (5 mm).

#### Apparatus

The experiment was presented on 22” monitor screens (Dell P2210, 1680 × 1050 at 60 Hz). Each of the computers was equipped with a RED-m remote eyetracker (SensoMotoric Instruments, Teltow, Germany) that recorded eye movements at a sampling rate of 120 Hz with a visual-angle accuracy of 0.5°, as reported by the manufacturer. Participants were calibrated in order to get an accuracy below 0.5°. When it was not achieved after three calibration attempts, validation results below 1° were accepted. The average validation accuracy was 0.70° ± 0.14°. The room in which the experiment was conducted consisted of 25 computers, each with one eye tracker.

#### Procedure

Participants received a short verbal introduction to the task during which they were informed that all people in the room would perform the same task. Participants were asked to work in silence and to avoid causing disturbance. Further detailed task instructions were presented on the screen, to ensure that participants understood the task, and to prevent participants from asking questions that would disturb others. In the group condition, participants sat adjacent to each other in a row of eight computers, separated from each other by approximately 100 cm. Participants sat at adjacent computers, regardless of the size of the group, to avoid differences in participant spacing due to differences in session sizes. Once all participants were successfully calibrated, participants were instructed to start the antisaccade task at the same time. The solitary participant recordings were performed in the same research facility. In both social conditions, the experimenter was in the room in a covert position that was not visible to the participants.

#### Data analysis

The start of the saccadic movements was detected by a custom version of an adaptive velocity-threshold-based event-detection algorithm (Nyström & Holmqvist, 2010). The saccade reaction time was defined as the time between the appearance of the stimulus and the start of the saccade away from the fixation point. Saccade onsets were determined by fitting a line to the part of the saccade where acceleration was positive and computing where this line intersected the abscissa. Trials where the starting fixation deviated more than 3° from the central fixation cross or where data samples were missing during the saccade were excluded. Saccades with a latency of less than 50 ms were considered anticipatory responses and excluded. Participants for whom less than 70% of the trials had a usable saccade under these criteria were excluded. Saccadic direction errors were coded as wrong when the first saccade after target presentation was in the wrong direction, that is, towards the stimulus during antisaccades.

As the distribution of latencies in response tasks is known to exhibit significant skew with a longer tail than a standard normal distribution (Carpenter & Williams, 1995; Reddi & Carpenter, 2000), the reciprocal of the saccadic reaction time, which does follow a Gaussian distribution (Noorani, 2014; Reddi, Asrress, & Carpenter, 2003) was used for all analyses. Participants are sometimes seen to make so-called ‘early responses’, saccades with extremely short latencies that do not belong to the main distribution of latencies (Carpenter & Williams, 1995; Munoz & Everling, 2004). Early responses were identified by fitting Carpenter’s LATER model (see ‘Computational modelling’) to the saccadic latency data. Specifically, when the reciprocals of the saccadic latencies are plotted on probit axes, the main population of responses falls along a straight line. The early responses are apparent from an inflection of the line at the early end of the distribution of latencies. Following Carpenter (Carpenter & Williams, 1995), per participant, the saccadic latencies in this space were fitted with two straight lines, one line with an unconstrained slope and intercept and one line whose intercept was fixed to the median of the latency distribution. Any saccades with latencies shorter than the intersection point of these two fitted lines were considered early and removed from further analysis. As identified by the LATER fit, there were scarce occurrences of early responses per participant in both the group (1.32* ± *0.30) and solitary conditions (1.07* ± *0.33).

We report *t* values and 95% confidence intervals for parameter estimates *b* arrived at through linear mixed effects modelling (Pinheiro & Bates, 2000) using R 3.0.1 and R package ‘nmle’, version 3.1-115. We report all the measures and variables.

#### Computational modelling

**Figure 1.**
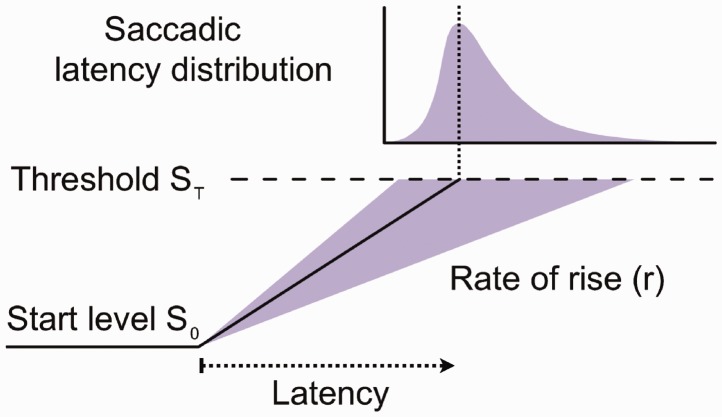
The LATER model. When a stimulus is presented, a decision signal *S* rises linearly from an initial level *S*_0_ at a rate *r*; when *S* reaches the threshold *S_T_*, a saccade is initiated. The rate of rise *r* obeys a Gaussian distribution, which gives a skewed distribution of latencies. Adapted from Gold & Shadlen, 2007.

To investigate whether social presence effects on reaction times are due to the accumulation of information signals or different decision thresholds, we fitted a LATER model to the saccadic reaction time data as seen in Figure 1. LATER is a decision model based on the concept that responses, such as saccades, follow a decision-making mechanism (Carpenter, 1981; Carpenter & Williams, 1995). When a stimulus is presented, the model predicts that a decision signal (*S*) increases from a baseline *S*_0_ at a certain rate *r* until it reaches a decision threshold *S_T_* that constitutes a criterion level (Carpenter, 2012; Carpenter & Williams, 1995; Gold & Shadlen, 2007; Reddi & Carpenter, 2000) where a decision to take action occurs, for instance, a saccadic movement towards a target. Specifically, the latency *t_i_* of any trial is given by $t_{i}=(S_{T}-S_{0})/r_{i}$. As this equation shows, different reaction times can result from varying either the difference between thresholds $S_{T}-S_{0}$ or the mean rate of rise *µ*. Since in the LATER model *r* is assumed to follow a Gaussian distribution, the latency *t* is said to follow a recinormal distribution and the reciprocal of the latency 1*/t* is normally distributed. As a consequence, a straight line is obtained if the cumulative distribution of the reciprocal of the latencies is plotted on a probit axis (a reciprobit plot), with median $(S_{T}-S_{0})/\mu$ and an intercept in the infinite-time axis at $\mu /\sigma \sqrt{2}$, where *σ* is the standard deviation of *r*.

**Figure 2.**
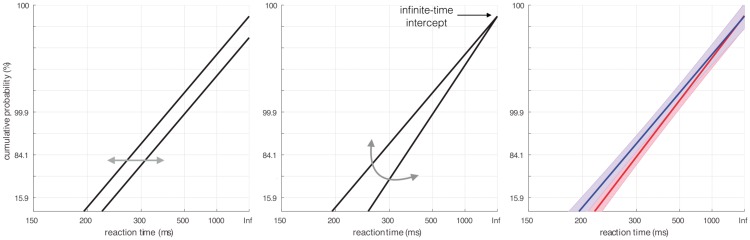
Reciprobit plots. The cumulative distributions of saccadic latencies are plotted on a probit scale with a reciprocal time axis. Left: an increase in the rate of rise of the decision signal would cause a parallel shift. Middle: an increase in *S_T_* will result in a swivelling of the line about the infinite-time axis. Right: the effect of the social conditions on the antisaccadic latency distributions. The group condition had a steeper line (red) than the solitary condition (blue), indicating an increase in the decision threshold. Note that for visualization purposes the *x*-axis is extended beyond the range of saccadic latency data used for this fit to show the intercept with the infinite time axis. The shaded areas represent the 95% confidence intervals.

As plotted in Figure 2, altering *S*_0_ or *S_T_* causes the line of the reciprobit to swivel about a fixed intercept on the infinite-time axis (see Carpenter & Williams, 1995; Reddi & Carpenter, 2000). In contrast, a change in *µ* causes a parallel shift of the reciprobit line (Reddi et al., 2003). Here we used this model to determine whether any changes in the saccadic latency we observed are due to a shift in the decision thresholds or due to a change in the rate of increase of the decision signal. Specifically, this was assessed by fitting a linear mixed effects model to the saccadic latency data of the two social conditions in probit spaces. For this fit, random intercepts and slopes for each participant were included in the model. The paucity of points in the tails of the distributions can make fit outcomes unstable. For this reason, and consistent with previous research (Harwood, Madelain, Krauzlis, & Wallman, 2008), we limited saccadic latencies included in this analysis to 400 ms. In contrast to Harwood et al. (2008), who used only 68% of the data (one SD surrounding the median), our criterion included 95% of the saccadic latency data in the analysis.

## Results

**Figure 3.**
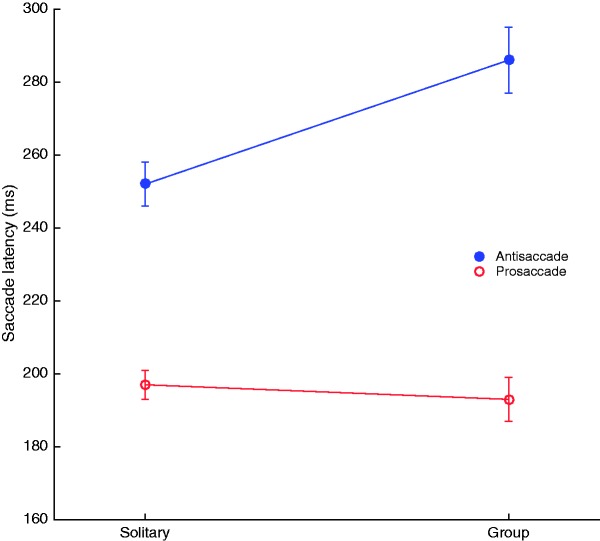
Pro- and antisaccadic latencies for the group and solitary conditions. Error bars indicate standard errors of the mean.

Average pro- and antisaccade latencies are plotted for the group and individual conditions in Figure 3. Correct antisaccades were consistently slower than prosaccades, in both the solitary condition (*b* = 1.15, 95% CI [0.84, 1.47], *t*(38) = 7.24, *p < *0.001) and the group condition (*b* = 1.74, 95% CI [1.43, 2.01], *t*(38) = 16.89, *p < *0.001). The antisaccade latency in the group condition (mean* ± *SEM: 286* ± *9 ms) was higher than in the solitary condition (252* ± *6 ms; *b* = 0.45, 95% CI [0.10, 0.78], *t*(38) = 2.73, *p* = 0.008, *d* = 0.92). In contrast, prosaccade latencies did not differ between the group (193* ± *6 ms) and solitary conditions (197* ± *4 ms; *b* = *−*0.18, 95% CI [*−*0.52, 0.16], *t*(38) = *−*1.06, *p* = 0.291, *d* = 0.18).

The average percentage of correct prosaccades was 99.5% in both social conditions; this low percentage of direction errors is expected as the prosaccade is a simple, automatic response. The average percentage of correct antisaccades was 81.4* ± *2.2% in the solitary condition and 80.3* ± *2.3% in the group condition, which is in line with the error rates usually found for adults in this task (Hutton & Ettinger, 2006). There were no differences in the numbers of incorrect antisaccades between conditions (*t*(38) = *−*0.46, *p* = 0.65).

Independent-samples *t*-tests were conducted to compare saccadic gain and the latency of corrective saccades between the group and the solitary conditions (see Table 1). However, the results indicated that there were no significant effects in either analyses (*p > *0.10 in all cases). The temporal resolution of the eyetrackers used did not allow us to assess differences in dynamic properties like peak saccadic velocities.

**Table 1.** Mean* ± *SE of Corrective Saccadic Latencies for Antisaccades and Saccadic Amplitudes (in Visual Angles) for Pro- and Antisaccades.

	Corrective Saccadic Latency (ms)	Prosaccadic Amplitude (°)	Antisaccadic Amplitude (°)
Solitary	167* ± *13	9.41* ± *0.10	9.52* ± *0.78
Group	184* ± *8	9.59* ± *0.13	9.32* ± *0.52

### Group size effect

**Figure 4.**
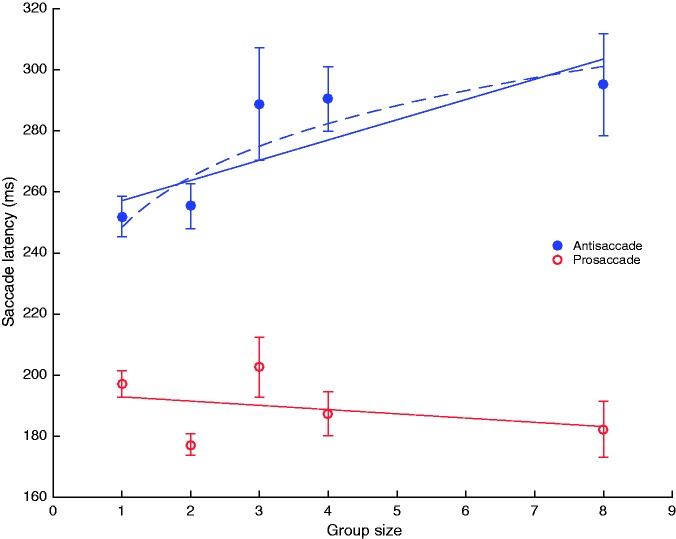
Antisaccade and prosaccade latencies plotted against group size. The dashed line represents a power law fit as predicted by Latane’s social impact theory. Error bars indicate standard errors.

To analyse in more detail what effect the presence of others has on antisaccade performance, we investigated how the group size, defined as the number of participants per session, influenced saccade latencies. Pro- and antisaccade latencies as a function of group size are plotted in Figure 4. Consistent with the results presented above, a mixed effects linear regression between the group sizes and the pro- and antisaccade latencies showed that antisaccade latencies increased with group size (*b* = *−*0.11, 95% CI [*−*0.20,* −*0.02], *t*(38) = *−*2.51, *p* = 0.016). The approximate parameter *b* indicates that the antisaccade latency should increase 7 ms for every additional participant in the session. Prosaccadic latencies, on the other hand, were not affected by the size of the group (*b* = 0.05, 95% CI [*−*0.03, 0.14], *t*(38) = 1.29, *p* = 0.204). The data furthermore suggest that the effect of group size on saccadic latency may be better described by a power law function (Figure 4), as proposed by Latane (1981). This issue is further discussed in the general discussion.

### Computational modelling

The LATER model allows investigation of the underlying cause of the increase in latencies found in the antisaccade task. Specifically, an increase in latency can have two underlying causes: an alteration in the mean rate of increase of the decision signal *µ* or a change in the difference between the decision thresholds $S_{T}-S_{0}$.

Figure 2 shows example reciprobit plots of saccadic latency data and illustrates how changes of the LATER model’s parameters are reflected in the reciprobit plot. A change in the mean rate of increase of the decision signal *µ* causes a parallel shift of the reciprobit line, which is reflected in a change of the intercept combined with a slope that does not change. In contrast, a change in the difference between the decision thresholds $S_{T}-S_{0}$ leads to a change of the slope but not of the intercept, which appears as a swivel of the reciprobit line about a fixed intercept on the time-infinity axis.

To assess whether the slope or the intercept of the reciprobit line differed between social conditions, we fitted the reciprocal of the saccadic latency data to a mixed effects model. Figure 2 shows the mean fit for each social condition. We found that while the intercept did not differ between the group and solitary conditions (*b* = *−*0.03, 95% CI [*−*0.86, 0.81], *t*(38) = *−*0.07, *p* = 0.94) the slope showed a trend towards being steeper in the group than in the solitary condition (*b* = 0.19, 95% CI [*−*0.02, 0.41], *t*(38) = 1.71, *p* = 0.08). One participant was a clear outlier in terms of the fit parameters from the model. The exclusion of only this participant makes the difference in the slopes of the reciprobits significant (*b* = 0.25, 95% CI [0.05, 0.45], *t*(37) = 2.51, *p* = 0.01), while the difference in intercepts remained insignificant (*b* = *−*0.19, 95% CI [*−*0.98, 0.60], *t*(37) = *−*0.49, *p* = 0.63). This participant was not an outlier in terms of the number of errors made or antisaccade latency and thus was kept in the analysis. However, the results strongly suggest that the longer latencies in the group condition can be attributed to a shift in the threshold parameter of the LATER model.

### Discussion

The results of Experiment 1 indicate that the antisaccade performance was impaired by the presence of others, while prosaccade performance was independent of the social context. The significant increase in the antisaccade latency together with no increase in direction errors of this response indicates that social presence influences the generation of voluntary behaviour rather than the inhibition control of automatic responses. These results are in line with previous studies assessing the influence of attentional load on saccadic control.

The distraction-conflict theory predicts that the attempt to attend to and process multiple inputs (i.e., both the task and the presence of others) increases attentional load (Baron, 1986). If this were the case, social presence ought to affect performance in line with what previous studies report about saccadic control and attentional load. Indeed, Stuyven et al. (2000) showed that increasing participants’ attentional load during the antisaccade task with a concurrent tapping task prolonged their antisaccade latency while prosaccades were not affected. A correct antisaccade requires the completion of two steps: an automatic prosaccade should be inhibited and a controlled saccade away from target should be generated (Olk & Kingstone, 2003). The delay in the antisaccade latencies in the group condition could be caused by an effect in one or both of these two antisaccade steps. A careful analysis suggests that an impairment in the capacity to suppress automatic prosaccades would have led to an increase in direction errors in the group condition, which was not observed. Consequently, we propose that social presence mainly influences participants’ capacity to generate controlled saccades rather than their ability to inhibit automatic saccades. This hypothesis is reinforced by the fact that Stuyven et al. (2000) showed that increased attentional load also prolonged the latency of controlled saccades even when there was no need to inhibit automatic behaviour as in the antisaccade.

The similarity between Stuyven et al. (2000) and the results of Experiment 1 suggests that the mechanism by which social presence influences performance is by increasing participants’ attentional load. The presence of coactors in the environment may lead participants to monitor their environment more than when they are alone. For instance, Muller et al. (2004) argued that individuals in a group are prone to social comparison. Therefore, by concurrently conducting the experimental task and monitoring the environment, participants would engage in a dual-task activity, increasing their attentional control requirements.

On the other hand, activation theory provides an alternative explanation to social influences, stating that social presence increases the likelihood of deploying automatic, dominant behaviour. In the case of the antisaccade task, prosaccades are clearly automatic responses (Roberts et al., 1994) and, therefore, we expected facilitation in the prosaccades, probably reflected by reduced latencies. Since no evidence for such facilitation was found, our data do not support the activation theory. It should be noted that automatic saccades are already rapid responses and it could be physiologically difficult to evidence facilitation by measuring a significant reduction of prosaccade latencies. However, further evidence that prosaccades were not facilitated is provided by the fact that there was no increase in antisaccade direction errors: if the likelihood of a prosaccade as the dominant response increased with social presence, participants should have made more errors trying to suppress prosaccades in favour of antisaccade responses.

Next, we analysed the saccadic latency distributions between social conditions with the LATER model. The results indicated that social presence led to delayed antisaccadic latencies in the group condition due to an increase in the decision thresholds rather than a change in the rate of rise of the decision signal. The decision thresholds relate to the criterion level (e.g., Carpenter & Williams, 1995; Reddi & Carpenter, 2000) at which saccades are executed. The saccadic procrastination, observed in the increased saccadic latency, accounts for the period when sensory information is integrated to decide where to attend and saccade to (Carpenter, 1981). This time window is part of a selection mechanism to control saccades when there are conflicting alternative targets (Schall & Hanes, 1993, 1998; Schall & Thompson, 1999). If participants are in a social environment and attention is allocated to more stimuli, a plausible compensatory mechanism might be to increase the decision threshold to allow more information to be integrated before the saccade away from target is initiated. In that sense, the increased decision threshold may allow for the correct inhibition of reflexive responses, even when the presence of coactors adds extra stimuli to the environment.

In order to investigate whether the same effect of social influence extends to other cognitively mediated responses, we conducted a second experiment that involved both saccades and manual responses.

## Experiment 2

Saccade responses are evoked faster than manual responses (Jaśkowski & Sobieralska, 2004) and there seem to be differences in the attentional control required for saccadic and manual responses (Ludwig & Gilchrist, 2002). In Experiment 2, we measured whether saccades are especially sensitive to social context, or whether manual responses are also influenced by the presence of coactors. We used a visual discrimination task that required participants to both make a saccade towards a target (targets were presented at varying distances in the periphery), and a manual response to indicate the orientation of that target. We measured two performance indicators: the saccadic latency of the saccade towards the target and the manual response time in reaction to the visual discrimination.

### Methods

#### Participants and design

As in Experiment 1, this experiment used a 2* × *2 mixed design (social condition vs response type, i.e. saccadic and manual responses). Experiment 2 was performed after Experiment 1. Therefore, the same participants took part and were assigned to the same conditions as in Experiment 1, and there was no opportunity for them to interact with one another between testing sessions. Two participants were excluded for having a high error rate (nearly chance performance); all other participants had error rates lower than 10%. Ten participants were excluded due to data loss (see ‘Data analysis’). The resulting numbers of participants included in the analysis were 21 in the group condition and 19 in the solitary condition.

#### Stimuli and material

A fixation cross was presented in the centre of the screen for a randomized interval of 60 to 140 screen refresh cycles (1000 ms to 2333 ms). Subsequently, a target was presented in a random position on the screen but at one of 10 possible distances from the centre. The presentation of the target occurred within a minimum radius of 5.4° and a maximum radius of 11.7°. The minimum radius was set in order to avoid recognition of the target through parafoveal vision and with the purpose of inducing a saccadic response. The space between the minimum and maximum radii was divided in eight sub-radii on which the target could be presented. Therefore, each trial required the generation of a saccade to one of 10 possible distances from the centre. There were 15 trials for each of the 10 radii, making a total of 150 trials.

The target consisted of a copyright symbol © displayed at a size of 1.4°. It could be presented as is or flipped horizontally, in which case the opening of the inner ‘c’ pointed towards the left. One of the two possible orientations was presented for every trial and the task consisted of reporting, as fast and accurately as possible, the orientation of the target, using the left and right arrows on the keyboard and using the dominant hand.

#### Apparatus

The research settings, hardware and software utilized for Experiment 2 are all the same as in Experiment 1.

#### Procedure

The procedure was similar to that of Experiment 1. A visual search task was added as a filler at the end, in order to ensure that all participants could finish the experimental task without disturbances.

#### Data analysis

The saccade onsets were calculated with the event detector and criteria described in Experiment 1. Only participants with more than 70% usable data were included in the analysis. Manual response times above 2.5 SD over the mean were excluded. All participants had error rates lower than 10%. The data were analysed with linear mixed models, allowing random slopes and intercepts for participants. As in Experiment 1, the reciprocal of the saccadic and manual reaction times was used for all analyses.

### Results

**Figure 5.**
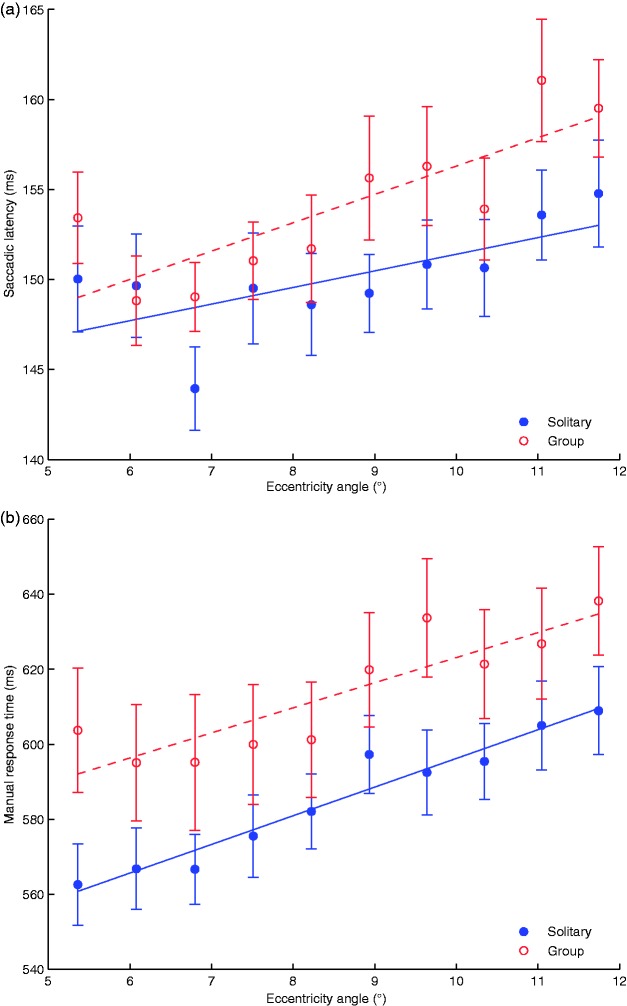
Saccade latencies and manual response times. (a) Saccadic reaction times in the group and solitary conditions were not significantly different. (b) Manual responses in the group condition tended to be slower across the entire range of eccentricities. Trend regression lines are shown for each of the conditions. Error bars indicate standard errors.

Figure 5(a) shows the mean saccadic latency as a function of target eccentricity for the group and solitary conditions. Linear mixed effects modelling revealed that while saccadic latencies varied with target eccentricity (*b* = *−*0.06, 95% CI [*−*0.09,* −*0.04], *t*(358) = *−*4.64, *p < *0.001), they did not differ between the two social conditions (*b* = *−*0.009, 95% CI [*−*0.44, 0.42], *t*(38) = *−*0.04, *p* = 0.96) and their interaction did not reach significance (*b* = 0.02, 95% CI [*−*0.01, 0.07], *t*(358) = 1.20, *p* = 0.23).

After making a saccade towards the target, participants had to report the orientation of the target. Manual response times are plotted in Figure 5(b) as a function of target eccentricity for the group and solitary conditions. Linear mixed effects modelling revealed that response latencies increased as a function of target eccentricity (*b* = *−*0.02, 95% CI [*−*0.02,* −*0.01], *t*(358) = 7.58, *p < *0.001). Manual response latency did not differ between the social conditions (*b* = 0.12, 95% CI [*−*0.02, 0.27], *t*(38) = 1.56, *p* = 0.11) and the interaction between target eccentricity and social condition did not reach significance (*b* = *−*0.004, 95% CI [*−*0.01, 0.003], *t*(358) = 1.07, *p* = 0.29).

#### Group size effect

Linear mixed effects modelling revealed that while saccadic latencies varied across target eccentricities, they did not differ as a function of group size (*b* = 0.02, 95% CI [*−*0.09, 0.14], *t*(38) = 0.39, *p* = 0.69). Furthermore, the interaction between target eccentricity and group size did not reach significance (*b* = *−*0.005, 95% CI [*−*0.018, 0.002], *t*(358) = 1.16, *p* = 0.24).

In Experiment 1, the average antisaccade latency increased with the number of coactors in the session. In order to see whether the effect on the manual response times could also be moderated by group size, the same analysis as in Experiment 1 was conducted. Linear mixed effects modelling revealed that there was a significant increase in latency as a function of group size (*b* = *−*0.04, 95% CI [*−*0.08,* −*0.02], *t*(38) = *−*2.22, *p* = 0.03), indicating that participants in bigger groups made slower manual responses. The interaction between target eccentricity and group size did not reach significance (*b* = 0.001, 95% CI [*−*0.001, 0.003], *t*(358) = 1.64, *p* = 0.11).

In summary, the saccade responses were independent of group size, while the manual response time increased significantly, with slower reaction times for bigger group sizes.

### Discussion

The choice reaction time task requires a reflexive response – the saccade towards the target – and a controlled component consisting of the discrimination of the stimulus. We measured saccadic and manual response times to differentiate between these simple and complex components of the task

The first step in the visual discrimination task, the saccade towards the target, is an automatic response analogous to a prosaccade where gaze is directed to an abruptly appearing target. In line with the results of Experiment 1, the saccadic latencies towards the visual target were not affected by the social environment. In contrast to Experiment 1 (antisaccades), the controlled responses in Experiment 2 (manual responses) were not clearly affected by the type of social context. On average, responses were 32 ms slower in the group condition than in the solitary condition, but this difference was only marginally significant. However, the size of the group had a significant effect on the manual reaction times, suggesting that social presence still elicited some influence on cognitively controlled manual responses.

The distraction-conflict theory supposes that the attentional conflict elicited by social presence focuses attention. Baron (1986) also extends this assumption to cues that are most visually central to the task. We assessed spatial narrowing through the saccadic latencies to targets presented at different eccentricities. We expected that a narrowing of the attentional window would prolong saccadic latencies towards targets presented farther away from centre. The results showed that social condition and eccentricity levels had no effect on saccadic latencies, suggesting that there was no such spatial narrowing. However, the eccentricity range analysed was from 5° to 12°; any potential focusing of attention on a more central area (*<* 5°) should be assessed in the future.

The effect of social influence may not only be restricted to a spatial narrowing of attention but also to other forms of attentional focusing such as the range of cue utilization or use of global and local information (e.g., Navon task). This offers interesting avenues for future research.

## General Discussion

We investigated how social presence affects saccadic and manual responses. Our main finding was that social presence had a significant effect on the generation of volitional saccades, but had no effect on automatic saccades. In contrast, a marginally significant effect was reported for controlled manual responses, but increasing group sizes moderated slower reaction times. Altogether, we assume that the group condition affects only controlled responses, presumably due to an increase in attentional load elicited by the presence of others. These results support the view that attentional processes are involved in the mediation of social influences. According to the LATER model analysis, the prolonged latencies can be explained in terms of an increase in the decision threshold, indicating that under social presence, more accumulation of evidence is required before a decision to trigger a controlled response is made.

The distraction-conflict theory states that social presence competes for cognitive and attentional resources with the task at hand. Since attentional capacity is limited, as demands for attention increase less spare capacity remains for other activities. Baron (1986) extended his theory not only to social stimuli but any stimuli that would withdraw attention from the main task. In fact, Stuyven et al. (2000) studied the effect of dual-task interference on the antisaccade task, specifically to assess the effect of attentional and cognitive load on automatic and controlled saccades. In those experiments, participants conducted an antisaccade task concurrently with a tapping task, but participants received different instructions regarding the importance of the secondary task. When the tapping task was less relevant to the overall performance, no effect on the antisaccades was observed. However, by instructing participants to keep a good tapping performance, the authors reported an increase in antisaccadic latency but not prosaccade latency. In other words, the amount of monitoring that participants allocated to the secondary task influenced the generation of only controlled saccades.

If there is an urge in individuals to monitor the social context when others are present, a similar analysis to that above can be performed when studying social influences. According to our results, this increased demand affected the generation of volitional responses, but did not impair the ability to suppress automatic behaviour when required, as evidenced by unchanged error rates. A relevant question now is *why* participants allocate attention to the environment when others are present. Previous studies suggest that the possibility for social comparison and adequacy to a social standard contributes to participants’ desire to monitor the social environment. Zajonc (1980) proposed that social presence increases individuals’ alertness because social presence adds uncertainty to the environment. The hypothesis was further developed by Guerin and Innes (1982), who suggested that task performance is affected when others cannot be visually monitored, or when others are unpredictable. Cottrell, Wack, Sekerak, and Rittle (1968) proposed that performance will change only when participants assume that they can be evaluated by others; however, this was later found not to be necessary for social facilitation–inhibition (Bond & Titus, 1983). Alternatively, Sanders et al. (1978) suggested that others provide social comparison information that can moderate social presence effects (Huguet et al., 1999; Muller et al., 2004). However, an implicit presence can also affect the deployment of social attention (Risko & Kingstone, 2009). Besides this, it was also proposed that certain personality traits moderate the general orientation of individuals towards the social environment (see Aiello & Douthitt, 2001, Strauss, 2002, and Uziel, 2007, for reviews). Carver and Scheier (1981) proposed in their feedback-loop model that social presence leads individuals to direct attention to the self in order to assess their own behaviour. This feedback-loop process may help participants to correct their behaviour and improve performance. However, self-attention can also interfere with the behavioural task and impair performance. This hypothesis also proposes an attentional mediation of social effects, but differs from the distraction-conflict theory in that attention is directed inwardly to the self rather than outwardly to the social environment. See Guerin (2010) for a review of social facilitation theories and findings.

As described, there are several potential reasons for why participants would engage in monitoring of the environment when others are present. An emerging question is *how* this attention to the environment is regulated. Attention to the environment could be driven by a volitional, controlled process or by a bottom-up, reflexive response. In this regard, the distraction-conflict theory refers to social presence as a distraction that competes for attention. The idea of distraction resembles a bottom-up type of influence. However, it could also be the case that individuals engage in an active monitoring of the social environment. Our view is that this process could be mediated by an interplay between volitional monitoring and bottom-up processes. Social presence might elicit a reflexive response that leads to the active monitoring of the environment. For instance, it could be that the urge for social comparison is a bottom-up process, but that the means by which individuals monitor the environment is achieved by top-down mechanisms. Further research should assess whether the potential causes mentioned above should be considered as bottom-up or top-down processes.

Dual-task experiments also showed that if the secondary task is excessively demanding, it can also impair the suppression of automatic saccades and increase direction errors. Roberts et al. (1994) studied the effect of dual tasks on the antisaccade with a concurrent task in a similar way to Stuyven et al. (2000), but with a more demanding interference task. Roberts et al. (1994) had participants listen to numbers, then perform an arithmetic calculation and verbalize the result. In contrast to Stuyven et al. (2000), Roberts et al. (1994) reported a significant increase in direction errors in the antisaccade task. Indeed, studies have shown that attentional depletion, caused by taxing the working memory capacity, affects individuals’ ability to suppress automatic saccades (Mitchell, Macrae, & Gilchrist, 2002). If interference disrupts the active maintenance of the task goals, diverted attention will lead to the deployment of automatic behaviour.

In a similar vein, cognitive distraction has also been found to interfere with the active maintenance of the task goals. For instance, Halliday and Carpenter (2010) explored the effects of cognitive distraction with a go/no-go saccadic task compared to a control group without distraction. This task again requires the inhibition of a saccade in the no-go trials. When participants were presented with bursts of pink noise concurrently with the task, neither the saccadic latency nor the direction error rates were affected. However, when participants performed a concurrent antonym verbal task, this led to higher direction error rates compared to the control group. Such results differ from the ones presented here in that inhibitory capacity was unaffected by our manipulation; rather, participants had prolonged response preparations. More examples where there is a deficit in response inhibition are provided by patients with attention-deficit hyperactivity disorder (ADHD). In a review, Munoz and Everling (2004) describe that what characterizes ADHD patients in the antisaccade task is increased direction errors, while correct antisaccades display normal reaction times, implying that they have no deficit in the capacity to initiate voluntary responses.

Thus, a reasonable interpretation would be that if you have a mild secondary task that you can handle, latencies but not direction errors are affected, but if the secondary task is very demanding, it can also affect direction errors. In our study, the effect of social presence specifically affected the generation of controlled saccades, suggesting that the coactors’ influence might be attributed to the engagement of participants in a mild dual-task activity by concurrently monitoring the environment.

The analysis conducted with the LATER model suggested that the response to social presence led to an increase in the decision thresholds. The functional significance of a change in the threshold parameters of the model has been assessed by Reddi et al. (2003) and Reddi and Carpenter (2000) in studies that investigated the effects of urgency and accuracy on saccadic responses. By asking participants to saccade either as fast or as accurately as possible towards a target, the authors showed that the decision threshold moved depending on the instructions. Faster response times were achieved by lowering the decision threshold, in which case the decision signal reached the criterion level to initiate a saccade in a shorter amount of time. In contrast, higher accuracy was achieved by increasing the decision threshold, so that more accumulation of the decision signal was required in order to initiate a saccade. If the environment becomes more uncertain with social presence, the selection mechanisms that decide where to allocate attention should also respond to such additional stimuli. In response, a plausible adaptation might be achieved by increasing the decision threshold that we found, allowing for more sensory integration before making the decision on where to saccade.

The other parameter of the model, the decision signal, characterizes the speed of acquisition of information about the stimulus (see Nouraei, de Pennington, Jones, & Carpenter, 2003; Reddi et al., 2003). We expected that this parameter could change if there were differences in the speed of processing elicited by the influence of the social environment (e.g., cognitive distraction that would slow down processing). However, no such result was found.

The comparison between solitary and group conditions showed that manual responses were only marginally affected by social presence. Such results contrast with the significant effect found for controlled saccades. Some differences between these responses might account for why social presence affected them differently. For instance, manual responses have significantly larger latencies than saccadic eye movements (Jaśkowski & Sobieralska, 2004; Ludwig & Gilchrist, 2002) and saccades have been shown to be more affected by attentional overload than manual responses are (Ludwig & Gilchrist, 2002). Therefore, saccadic responses should better reflect early processes of attention selection, while the longer latencies of the manual responses might allow for more sensory integration before an action is taken. Saccades occur at a rate of two to three times per second in humans (Holmqvist et al., 2011), while manual responses in reaction to visual stimuli are scarce in comparison. Additionally, in the antisaccade task, the correct volitional response is in competition with a more reflexive – but erroneous – prosaccade. In contrast, no inhibitory control is required in the manual responses of Experiment 2. These additional attentional demands in the antisaccade task may account for why such responses are more sensitive to, for example, attentional load, than manual responses when others are present.

In both of our experiments, the number of people present in the recording session had a significant effect on performance change. A larger number of coactors may reinforce individuals’ awareness of the social presence and, hence, moderate the extent to which attention is allocated to the environment at the expense of attention used for the experimental task. Latane (1981) proposed that a crowd (or an audience) of a determined size (*N*) will have an *impact* (*I*) on an individual’s behaviour that can be described as a power law function of the form $I={\mathrm{sN}}^{t}$, with *s* being a constant and the exponent *t* being lower than one. The function describes an increase in social impact with group size that eventually flattens out with bigger crowds. Although the theory prediction does not always hold (Argo, Dahl, & Manchanda, 2005), our results seem to be consistent with Latane’s social impact theory. The relationship between group size and the increase in the antisaccade latencies seems to flatten out (Figure 4). The best-fitting power function calculated accounts for 59% of the variance in means (better than the 43% best linear fit). It is worth noting that Latane’s theory relates to crowd or audience conditions, whereas we used a coaction manipulation. Therefore, it is advisable to further study the role of group size in more dedicated experiments.

The present study focused on how the presence of coactors influenced saccadic and manual responses. Participants in our experiments were not required to compete and did not receive explicit interaction instructions. In this sense, our results constitute a baseline measure of how attentional control is affected by conditions of coaction. For this purpose, eye movements proved to be a successful novel strategy for the study of the attentional effects of social influences. This should open up the door to future studies investigating participants’ attentional control under different manipulations (e.g., competition, evaluation, cooperation) that may lead to novel findings.

## Conclusions

In conclusion, the present study provides evidence for an attentional view of coaction effects. Even when participants are not directly interacting with each other, the presence of coactors seems to consume attentional resources that affect controlled saccadic responses but not automatic saccades. Manual responses were not affected by the social context, probably due to the fact that saccades can be evoked more rapidly than manual responses. However, the number of coactors in the group moderated the change in performance for both controlled saccadic and manual responses. The analysis of latency distributions with the LATER model indicates that the delayed saccadic latencies in the group condition are due to an increase in the decision threshold of when to initiate a response.
